# The Subtle Signs of Scurvy: A Paediatric Case Misleading Clinicians

**DOI:** 10.7759/cureus.73032

**Published:** 2024-11-05

**Authors:** Melvin Lee Qiyu, Amna Ahmed, Tom Dawson

**Affiliations:** 1 Paediatric Respiratory Medicine, Birmingham Children Hospital, Birmingham, GBR; 2 Paediatrics and Child Health, Worcester Royal Hospital, Worcester, GBR; 3 General Paediatrics, Worcestershire Acute Hospitals NHS Trust, Worcestershire, GBR

**Keywords:** bone edema, muskuloskeletal mri, peripheral weakness, uncommon scurvy manifestation, vitamin c in health and diseases

## Abstract

Scurvy, arising from vitamin C deficiency, remains relevant despite historical declines. Scurvy commonly presents with severe leg pain, reluctance to walk, and limping. Other symptoms include gingival bleeding, hypertrophy, and ecchymoses. Due to its rarity in the paediatric population, vitamin C deficiency poses a diagnostic challenge.

This case report details a 3-year-old boy presenting with a four weeks' history of being unable to bear weight and fine petechial rashes on lower limbs, initially evaluated for an injury. With no apparent fractures from the X-rays, further investigations were carried out, including MRI Short Tau Inversion Recovery (STIR). The MRI STIR results revealed bone and soft tissue oedema, prompting concerns about systemic disorders. Abnormal blood results, coupled with comorbidities such as suspected autistic spectrum disorder, triggered collaboration among paediatric, orthopaedic, and haematology/oncology specialists. Extensive imaging and consultations unveiled a diagnosis of scurvy, illustrating the challenges in recognizing this vitamin deficiency amid overlapping symptoms with serious conditions. The interdisciplinary team initiated treatment with oral ascorbic acid and analgesia, addressing the patient's reluctance to consume a varied diet. Parental decline for measuring ascorbic acid levels added complexity. Following a multi-disciplinary approach involving paediatric dietitians and physiotherapists, the patient exhibited positive progress, emphasizing the importance of tailored interventions.

This case highlights the diagnostic challenges posed by scurvy's diverse clinical presentations and the imperative for healthcare providers to recognize its potential, especially in cases involving musculoskeletal symptoms. The enduring relevance of scurvy in contemporary healthcare underscores the need for heightened awareness, interdisciplinary collaboration, and ongoing monitoring, emphasizing both short-term treatment and long-term preventive measures through dietary habits.

## Introduction

Scurvy stands as one of the earliest recognized diseases resulting from a deficiency in vitamin C. The symptoms of this condition were initially recorded by ancient Egyptian medical practitioners in 1550 BC, who recommended treatment with onions and vegetables [1]. In the 1700s, James Lind, a surgeon in the British Royal Navy, made a noteworthy breakthrough by discovering that consuming lemons and oranges could relieve the symptoms of vitamin C deficiency. Humans do not possess the active form of the enzyme L-gulonolactone oxidase necessary for the synthesis of ascorbic acid thus it can only be obtained from dietary sources or supplements [1].

The prevalence of this condition has significantly diminished over time, thanks to the modernization of society and the increased accessibility of vitamin C-rich foods [1]. The fact that scurvy can still occur in our current generation should not be overlooked [2]. Despite the abundant sources of vitamin C, instances of this nutritional deficiency ailment can persist, serving as a reminder that vigilance regarding proper nutrition remains essential. According to the 2003-2004 cross-sectional study conducted by the National Health and Nutritional Examination Survey in the United States, the prevalence of vitamin C deficiency in the overall population, encompassing individuals aged 6 years and older, was approximately 7.1%. Breaking down the data further, the study revealed that the prevalence of vitamin C deficiency was 1.6% in children aged 6-11 years and less than 4% in adolescents [3].

The prevalence of scurvy is intricately linked to the socioeconomic status of a given region. An Indian study exemplifies this association, indicating that the standardized prevalence of vitamin C deficiency was 73.9% in North India and 45.7% in South India [4]. Scurvy typically manifests clinically with musculoskeletal complaints, with severe pain in the lower limbs being reported in 88% of cases. Additionally, patients may exhibit a reluctance to walk (73%) and limping (31%). Other common presentations involve gingival bleeding (43%), gingival hypertrophy (27%), and ecchymoses (39%). Less common signs and symptoms include pyrexia (17%), nose bleeding (5%), perifollicular hemorrhage (7%), and irritability (10%) [5]. 

This article was previously posted to the Research Square preprint server on May 2, 2024. 

## Case presentation

A 3-year-old boy presented to the emergency department following a one-week history of being unable to bear weight. He was seen by the orthopaedic team due to concern about a potential fracture of his lower limbs following a twist and fall from standing one week earlier. Upon examination, there was no evident swelling or redness in both legs, yet the patient was reluctant to bear weight. Irritability was noted upon palpation of the lower back and hips, with an otherwise normal neurological examination. There were no signs of ecchymosis, oral mucosal bleeding, or gingivitis observed. Some fine petechial rash on the lower limbs was noted. He weighed 12.8 kg and was in the 25th percentile for his age.

**Figure 1:**
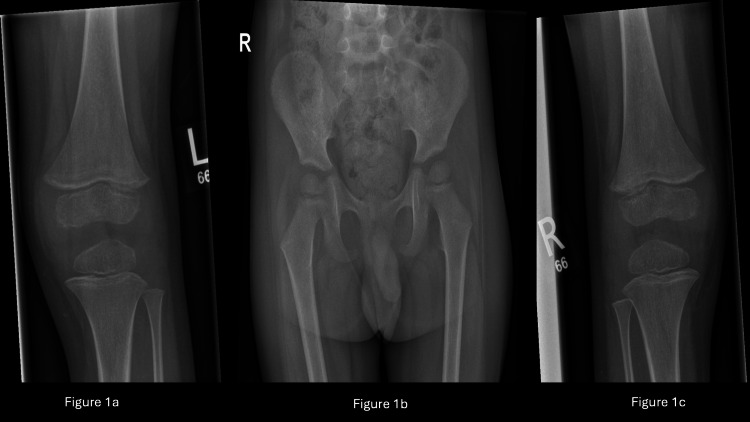
X-rays of pelvis and bilateral knees during initial presentation to Emergency Department Figure 1a is the left knee, Figure 1b is the pelvis and Figure 1c is the right knee. No fractures or bone lesions were observed. The hip joints appear symmetrical and no effusion is present in the knee joints.

Initial X-rays (Figure 1) of both lower limbs, pelvis, and knees showed no apparent fractures or effusions, and inflammatory markers were within normal limits, with a C-reactive protein (CRP) of 5 and normal white cell counts of 8x10^9^/L. In the patient's medical history, there was a suspicion of autistic spectrum disorder (ASD) noted by the general practitioner (GP), and a referral was made to the Child and Adolescent Mental Health Service (CAMHS) for further evaluation but was on the waiting list. The patient was managed conservatively as soft tissue injury with painkillers and follow-ups in the orthopaedic clinic. 

**Figure 2:**
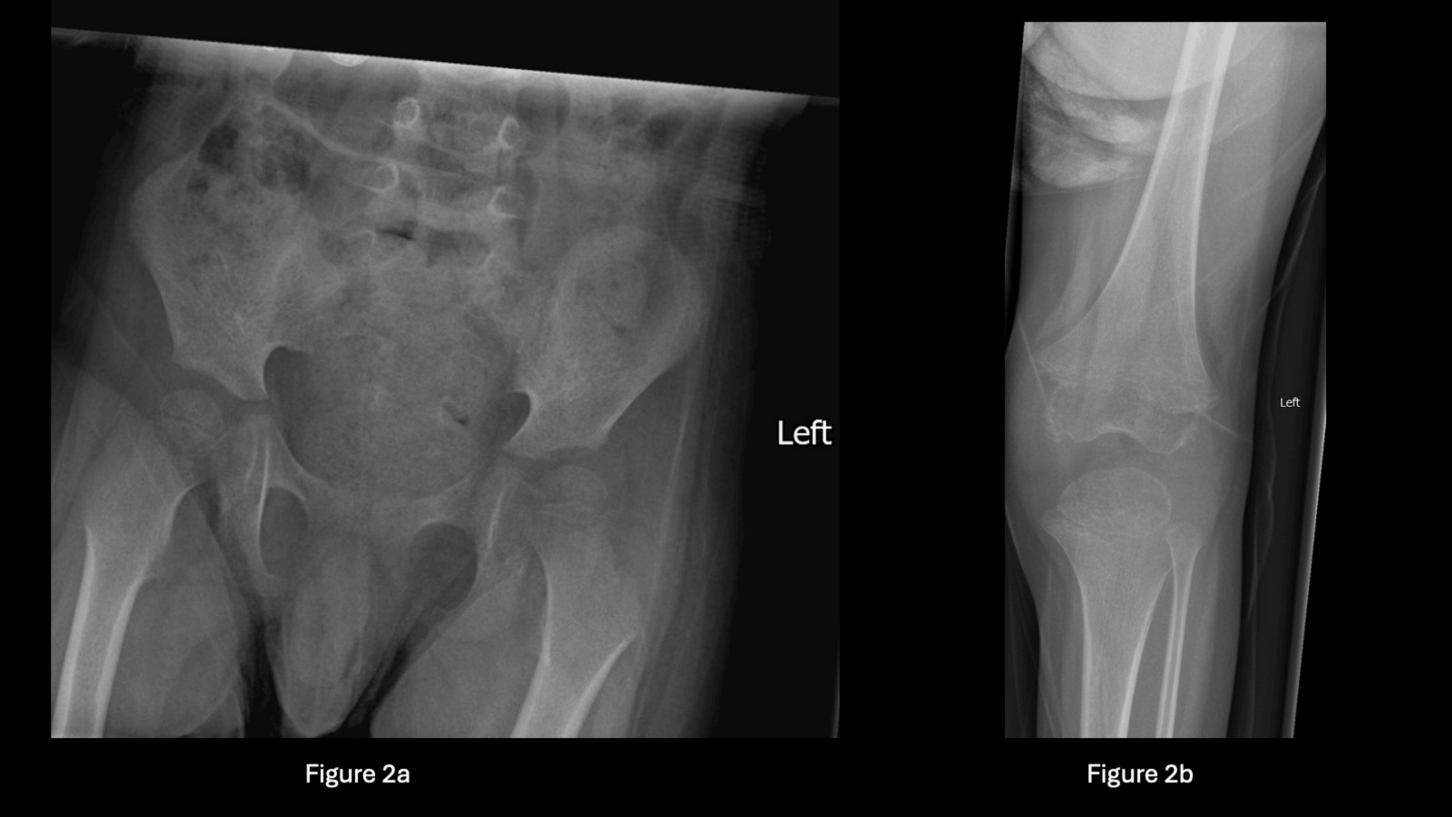
X-rays of pelvis and left knee on follow up in 3 weeks' time Figure 2a is the pelvis and Figure 2b is the left knee. No obvious abnormality was noticed on the pelvis. However, irregularities were seen in the left knee epiphysis.

The patient continued to be monitored in the orthopaedic clinic over subsequent weeks, with a follow-up set of X-rays for the pelvis and bilateral knees (Figure 2) conducted on the third week, revealing irregularities in the left knee epiphysis. Because of the persistent non-weight-bearing status for a total of four weeks, an MRI of the entire spine, pelvis, hips, and knees under general anaesthesia was arranged. He was then admitted under the General Paediatric and orthopaedic teams for joint care at that stage. Further history was revealed during this admission, including the patient's poor diet intake, with primary consumption of plain bread with butter and cow's milk. 

**Figure 3:**
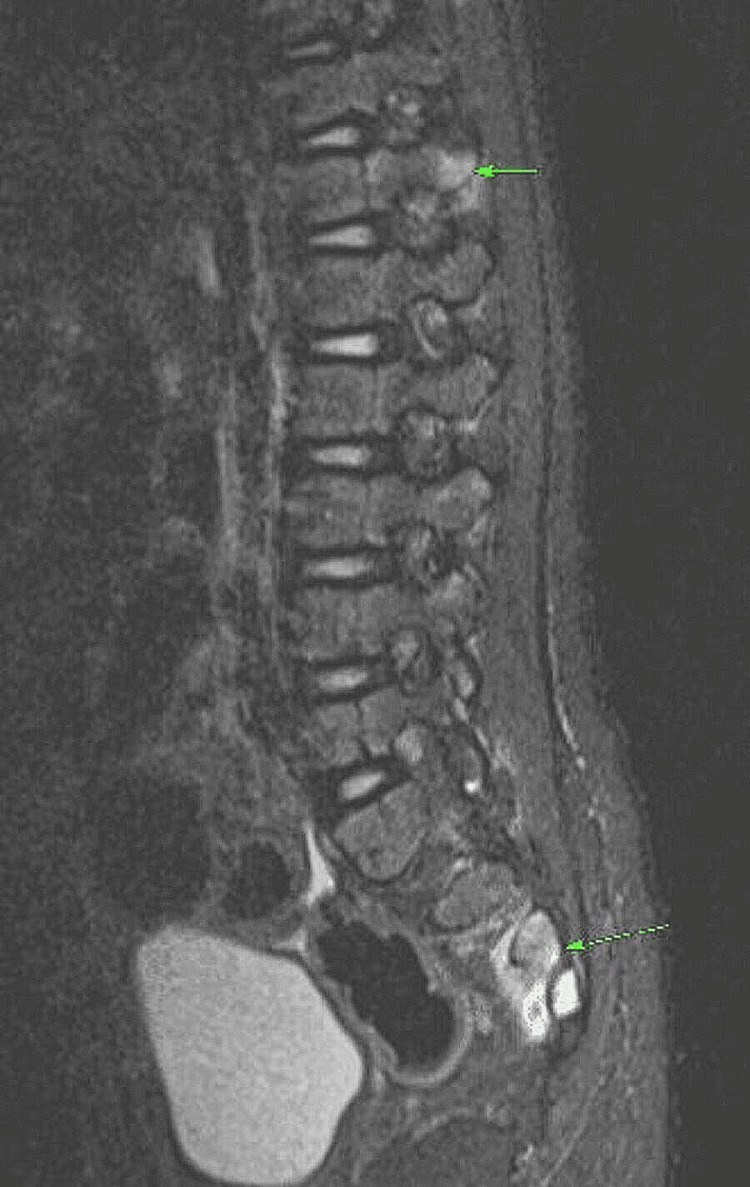
MRI of lumbar spine. Mild bone oedema is seen in right transverse processes of T12, L3, L4, L5, periarticular region of sacrum and lower sacrum bodies (particular adjoining lateral cortices). Areas marked by green arrows are bone oedema.

The MRI scan identified mild bone oedema at the right transverse processes of T12, L3, L4 and L5, particularly along adjoining lateral cortices, accompanied by subtle soft tissue oedema (Figure 3). The scan results prompted consideration of various differential diagnoses, including systemic abnormalities related to metabolic conditions, inflammatory processes, or malignancy.

Blood results were noted, including 25-Hydroxy Vitamin D at 48.6 nmol/L, C-reactive protein at 19, erythrocyte sedimentation rate at 43 mm/hr, red cell microcytosis on the blood film and lactate dehydrogenase (LDH) at 132 U/L (Table 1). In response to these findings, the patient was initiated on cholecalciferol at 600 units once daily as a maintenance dose for vitamin D.

**Table 1:** Summary of laboratory blood test results. Abnormal results include low 25-Hydroxy Vitamin D, and raised C-reactive protein, erythrocyte sedimentation rate, and lactate dehydrogenase.

Laboratory investigation	Results	Normal range
Alanine Aminotransferase	9	9-23 (U/L)
Alkaline Phosphatase	179	132-315 (U/L)
Total bilirubin	10	5-21 (umol/L)
Calcium (Ca)	2.64	2.2-2.7 (mmol/L)
Adjusted calcium	2.66	2.2-2.7 (mmol/L)
Magnesium (Mg)	0.81	0.7-1 (mmol/L)
Albumin	43	30-50 (g/L)
C-reactive protein	19	0-5 (mg/L)
Total protein	71	60-80 (g/L)
Sodium (Na)	136	133-146 (mEq/L)
Potassium (K)	4.7	3.5-5.3 (mEq/L)
Urea	4.5	2.5-6.5 (mmol/L)
Creatinine	28	15-33 (umol/L)
Phosphate	1.28	0.9-1.8 (mmol/L)
Erythrocyte Sedimentation Rate	43	1-10 (mmm/hr)
Haemoglobin (Hb)	12.8	11.0-14.0 (g/dL)
White Cell Count	8.3	5.5-15.5 x (109 /L)
Platelet	351	200-450 x (109 /L)
25-hydroxy vitamin D	48.6	50-150 (nmol/L)
Parathyroid hormone	1.3	1.3-9.3 (pmol/L)
Vitamin B12	491	133-675 (pg/mL)
Folate	5.7	3.1-19.9 (ng/mL)
Total Creatine Kinase	51	0-320 (U/L)
Ferritin	30	23.9-336.2 (ng/mL)
Thyroxine (T4)	12.2	9.5-17.8 (mcg/dL)
Thyroid-stimulating hormone	1.1	0.79-5.85 (mIU/L)
Lactate Dehydrogenase (LDH)	132	159-266 (U/L)
Uric acid	254	200-430 (mg/dL)

**Figure 4:**
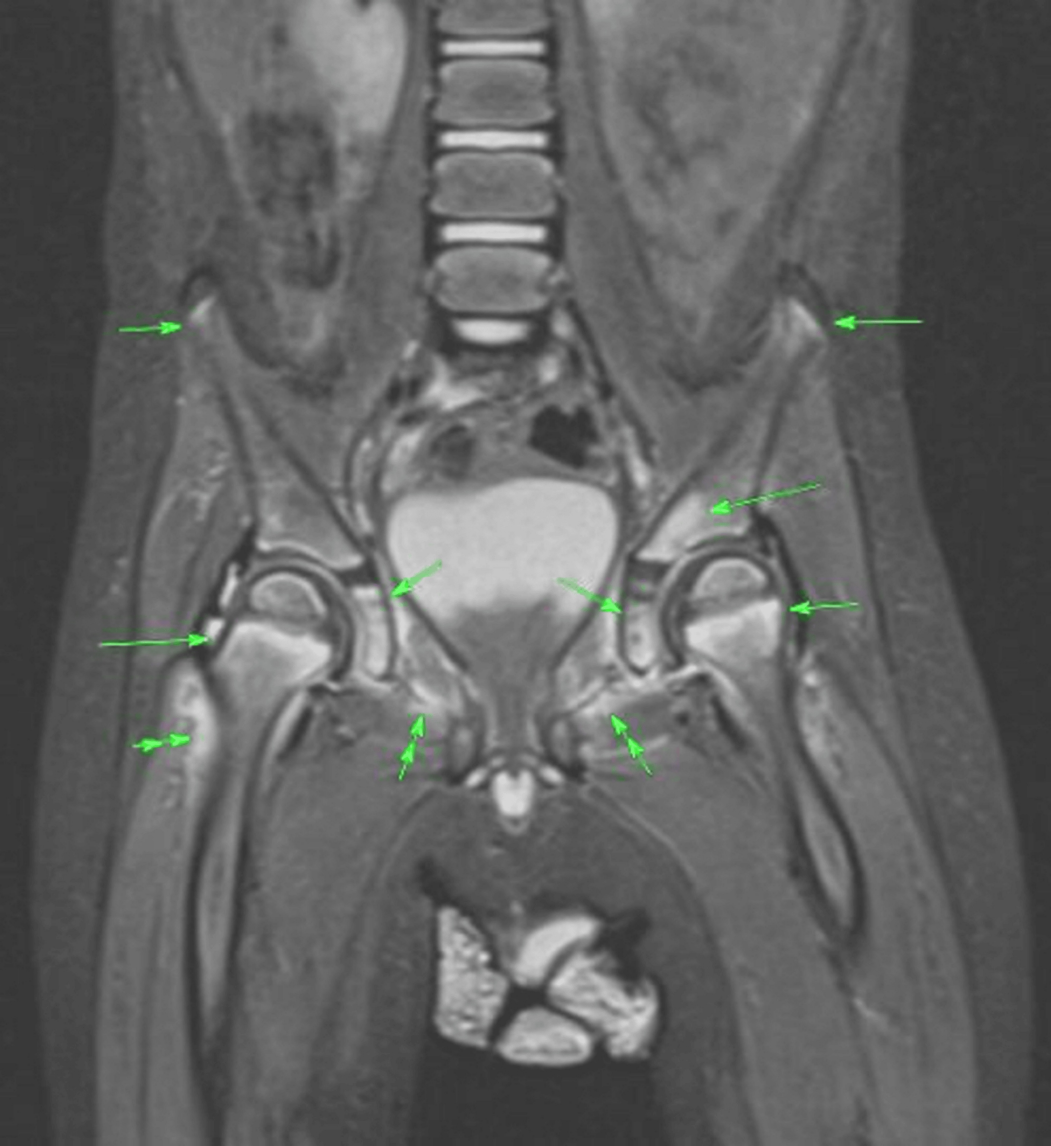
MRI STIR of bilateral hips. Symmetrical bone marrow oedema involving multiple metaphysis with associated peri-osseus soft tissue and muscle oedema are also noted. Area marked by single green arrows are bone oedema and double green arrows are muscular and soft tissue oedema. STIR: Short Tau Inversion Recovery

The patient's case was then discussed with the tertiary paediatric haematology/oncology team due to concerns about a potential malignancy and accepted for further investigation. A whole-body MRI Short Tau Inversion Recovery (STIR) was completed, revealing increased signal intensity within the metaphyses of all long bones, particularly around both acetabulum and within tarsal bones. Symmetrical metaphyseal oedema was observed in several joints, including the hips, knees, and wrists. Bilateral symmetrical muscle oedema around the pelvis, involving the adductor and obturator muscles was also visible (Figure 4).

Given the pronounced metaphyseal changes, an underlying systemic disorder was deemed the most probable cause. Additional investigations were initiated due to this atypical presentation, including microscopy of bone marrow and bone marrow culture following the MRI results. Microscopic examination of the bone marrow did not reveal any signs of leukaemia, and the culture also returned negative results, effectively ruling out infective causes of bone marrow involvement and chronic recurrent multifocal osteomyelitis (CRMO).

A multi-disciplinary team (MDT) meeting was convened, involving specialists from haematology/oncology, dermatology, metabolic, rheumatology, and radiology, to discuss the case. The clinical presentation, negative laboratory results, and imaging findings collectively led to the impression of scurvy. To address this, a trial of oral ascorbic acid at a dosage of 250 mg once daily was initiated. Additionally, to achieve better pain control, a higher dose of ibuprofen at 30 mg/kg/day was prescribed for a duration of two weeks, accompanied by 10 mg once daily Omperazole for stomach protection. However, the ascorbic acid level was not measured due to familial refusal. The patient was also referred to a paediatric dietitian for the development of a tailored dietary plan, considering his poor dietary intake. In parallel, a referral to a physiotherapist was made to address and improve the patient's limited mobility.

He was transferred to a regional paediatric ward to continue physiotherapy and engage with dietetic support for introducing a variable of food. Following some improvement in his mobility, he was discharged the following week. At one-month follow-up in the dietitian clinic, there was positive progress and his mother was satisfied with his increased weight. Successful incorporation of eggs, milk, yoghurt, and a variety of fruits into his routine diet was achieved. There was still a limited range of dietary vegetables, so the introduction of broccoli, spinach, and carrots was targeted.

During the clinic visit, it was noted that he had been fully mobile for the past week without the need for further painkillers. The plan included a dietetic clinic follow-up in three months and a paediatric medical review was scheduled for two months' time. Overall, the patient's recovery and dietary adjustments showed encouraging signs and ongoing monitoring was planned to ensure continued progress.

## Discussion

Diagnosing scurvy can pose challenges since individuals with the condition may not consistently exhibit typical features such as lethargy, bleeding gum, bruises, and anaemia. Additionally, scurvy symptoms can overlap with those of more serious conditions, such as leukaemia, bone malignancies, infectious diseases, and rheumatological disorders, presenting with signs like bleeding, lethargy, and anaemia. Consequently, this can result in delayed diagnosis and necessitate extensive investigations [6]. In a narrative review of children diagnosed with vitamin C deficiency between January 2000 and December 2021, a substantial number of patients, specifically 76% (127 out of 166), showed comorbidities associated with an increased risk of vitamin C deficiency [5].

The prevalent comorbidities included neurodevelopmental disorders such as autism, anorexia, cerebral palsy, and developmental delay, collectively accounting for 29% of cases [5]. Additionally, various other risk factors were identified, encompassing haematological disorders, multiple malnutrition, and other related conditions. It is a common occurrence for patients with scurvy to initially be referred to the orthopaedic team, often due to the nature of their presenting history. Therefore, both paediatric and orthopaedic teams must heighten awareness and develop the ability to recognize the common bony features of scurvy, such as osteodynia, inability to bear weight, and a dense metaphyseal line visible on X-ray [7]. A retrospective study conducted in the United States revealed that a considerable number of patients diagnosed with scurvy had undergone extensive imaging, including multiple series of MRI scans [8]. The majority of scurvy diagnoses rely on clinical judgment but can be overlooked or delayed due to its low prevalence in the modern era. The dosage of vitamin C supplements for treating scurvy varies depending on the severity of the disease and age groups. For children, the recommended dosage is 300 mg/day, while for adults, it ranges from 500 to 1000 mg/day [1]. The treatment duration typically spans 1 to 3 months or until all symptoms have completely resolved [1]. It is highly recommended to continue consuming regular fruits and vegetables rich in vitamin C to maintain sufficient levels in the body and prevent recurrence. This approach emphasizes both short-term treatment and long-term preventive measures through dietary habits [1].

## Conclusions

This case highlights the importance of considering scurvy as a potential diagnosis, especially in atypical presentations, such as the case of a toddler with musculoskeletal complaints and a history of non-weight bearing. The patient's comorbidities, including suspected autistic spectrum disorder, played a role in the delayed recognition of scurvy. This case serves as a reminder that scurvy, despite being historically associated with sailors and explorers, can still occur in contemporary society. Recognizing the diverse clinical presentations, addressing potential comorbidities, and promoting awareness among healthcare providers are essential for timely diagnosis and effective management of scurvy. Continued monitoring and preventive measures, including dietary interventions, are crucial to ensuring sustained improvement and preventing recurrence in patients at risk for vitamin C deficiency.
